# Vascular Foramina of Dry Human Talus: A Morphological Study

**DOI:** 10.7759/cureus.59060

**Published:** 2024-04-26

**Authors:** Mehmet Ülkir, Bahattin Paslı

**Affiliations:** 1 Department of Anatomy, Hacettepe University, Ankara, TUR

**Keywords:** foraminal index, anatomy, morphology, vascular foramina, talus

## Abstract

Background

The talus is the second largest tarsal bone and makes the osseous link between the leg and foot region. The branches of the dorsalis pedis, posterior tibial, and peroneal arteries enter vascular foramina (VF) on the various surfaces of the talus and provide intraosseous blood supply. Understanding the morphology and morphometry of VF might be helpful in reducing the risk of vascular injury associated with surgical interventions to the talus.

Aim and objectives

The purpose of this study is to contribute reference data for the morphology of VF of talus in a sample from Türkiye.

Materials and methods

This study was performed on 62 dry talus samples from Türkiye. The number, location, size, and foraminal index of the VF were evaluated on each talus. The total and medial surface lengths, distances of the closest and furthest foramina on the inferior surface, and distances of the closest and furthest foramina on medial surface were measured.

Results

No VF was detected on articular surfaces and the head of the talus. The majority of VF (1754; 81.17%) were detected on the neck, and 708 (40.36%) were located on the inferior surface of the neck. On the body, VF was mostly detected on the medial surface (233; 57.25%). The mean foraminal indices of the closest and furthest foramina on the inferior surface were 38.85% and 77.89%, respectively. The mean foraminal index of the closest foramina on the medial surface was 33.52%, and the furthest foramina on the medial surface was 63.91%. The total number of VF on 62 tali was determined as 2161. The majority (1521; 70.38%) of the size of VF was ≥0.6 mm. The mean total length was 55.14±4.69 mm, and the medial surface length was 49.18±4.18 mm.

Conclusion

Knowing the morphologic and morphometric properties of the VF gains importance during surgical interventions to the talus to reduce vascular damage. According to our results, lateral approaches to the talus may be safer than other approaches. To our knowledge, there is no study about the morphology of VF of the talus in Türkiye samples. We believe that the results of this study will provide reference data for morphology and morphometry of VF of talus.

## Introduction

The talus is the second largest tarsal bone and makes an osseous link between the leg and foot region. It is also named in other animals as "astragalus" [1]. Because of its weight-bearing role, it transmits the weight from the tibia to the heads of the metatarsals anteriorly and calcaneus posteriorly [2]. The talus has three anatomical parts (head, neck, and body) and is only tarsal bone without a musculotendinous attachment [3,4]. The extraosseous blood supply of the talus is provided by three primary sources, including dorsalis pedis, posterior tibial, and peroneal arteries [5]. Vascular foramina (VF) is observed elsewhere in irregular bones and on the diaphysis in long bones [6,7]. The branches of these arteries enter to VF on the various surfaces of the talus and provide an intraosseous blood supply [8]. These branches anastomose in the sinus tarsi, they may rupture in cases such as bone fracture, and this may cause avascular necrosis [9].

Understanding the morphology and morphometry of VF might be helpful in reducing the risk of vascular injury associated with surgical interventions to the talus. To our knowledge, there is no study about the VF of the talus in Türkiye samples. The purpose of this study is to contribute reference data for the morphology of VF of talus.

## Materials and methods

Data collection

This study was performed on 86 dry tali samples from Türkiye, which were obtained from the Department of Anatomy, Faculty of Medicine, Hacettepe University. Twenty-four tali with gross pathologic changes and damages were excluded from the study. Finally, 62 dried tali (26 right, 36 left) were examined. The age and sex of the bones were unknown. The side estimation of the tali was done using some anatomical landmarks (lateral process, shapes of articular surfaces for medial (comma shaped), and lateral malleolus (triangular shaped) [3]. Ethical approval was obtained from the non-interventional Clinical Research Ethics Committee of Hacettepe University (date: 06/06/2023, number: GO 23/490).

**Figure 1:**
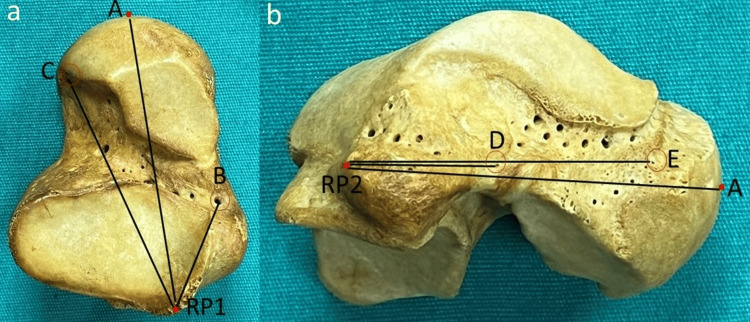
Demonstration of reference points Image a: A - the most anterior point of the head of the talus; B - the closest foramina on the inferior surface; C - the furthest foramina on the inferior surface; RP1 - the most posterior point on the lateral tubercle of the posterior process Image b: A - the most anterior point of the head of the talus; D - the closest foramina on the medial surface; E - the furthest foramina on the medial surface; RP2 - the most posterior point on the medial tubercle of the posterior process

Firstly, the magnifying hand lens was used to detect the VF macroscopically on the surfaces of the head, neck, and body of the tali. The definition of the VF was done according to the presence of a well-defined canal and groove. A gauge size 26 hypodermic needle with a diameter of 0.6 mm was used to confirm the patency of the foramen. The number, location, and size of the VF were evaluated. The VF was classified as large (≥0.6 mm) if it allowed the needle to pass and small (<0.6 mm) if it did not. The calcaneal articular facets of the talus were classified into three types according to Boyan et al.; type A - anterior, middle, and posterior facets are separated; type B - anterior, middle facets are fused, and posterior facet is separated; type C - anterior, middle and posterior facets are fused [10]. The reference points (RP) were determined to be the most posterior points on the lateral (RP1) and medial (RP2) tubercles of the posterior process. On the inferior surface and medial surface of the talus, the closest and furthest foramina from the RP1 and RP2 were noted, respectively (Figure 1).

The following parameters were measured: 1) total length (TL) - the distance between the RP1 and the most anterior point of the head of the talus (A-RP1); 2) medial surface length (ML) - the distance between the RP2 and the most anterior point of the head of the talus (A-RP2); 3) the closest foramina on the inferior surface (CFI) - the distance between the RP1 and the closest foramina on the inferior surface (B-RP1); 4) the furthest foramina on the inferior surface (FFI) - the distance between the RP1 and the furthest foramina on the inferior surface (C-RP1); 5) the closest foramina on the medial surface (CFM) - the distance between the RP2 and the closest foramina on the medial surface (D-RP2); 6) the furthest foramina on the medial surface (FFM) - the distance between the RP2 and the furthest foramina on the medial surface (E-RP2); 7) the foraminal index 1 (FI1) - CFI/TL × 100; 8) the foraminal index 2 (FI2) - FFI/TL × 100; 9) the foraminal index 3 (FI3) - CFM/ML × 100; 10) the foraminal index 4 (FI4) - FFM/ML × 100.

**Figure 2:**
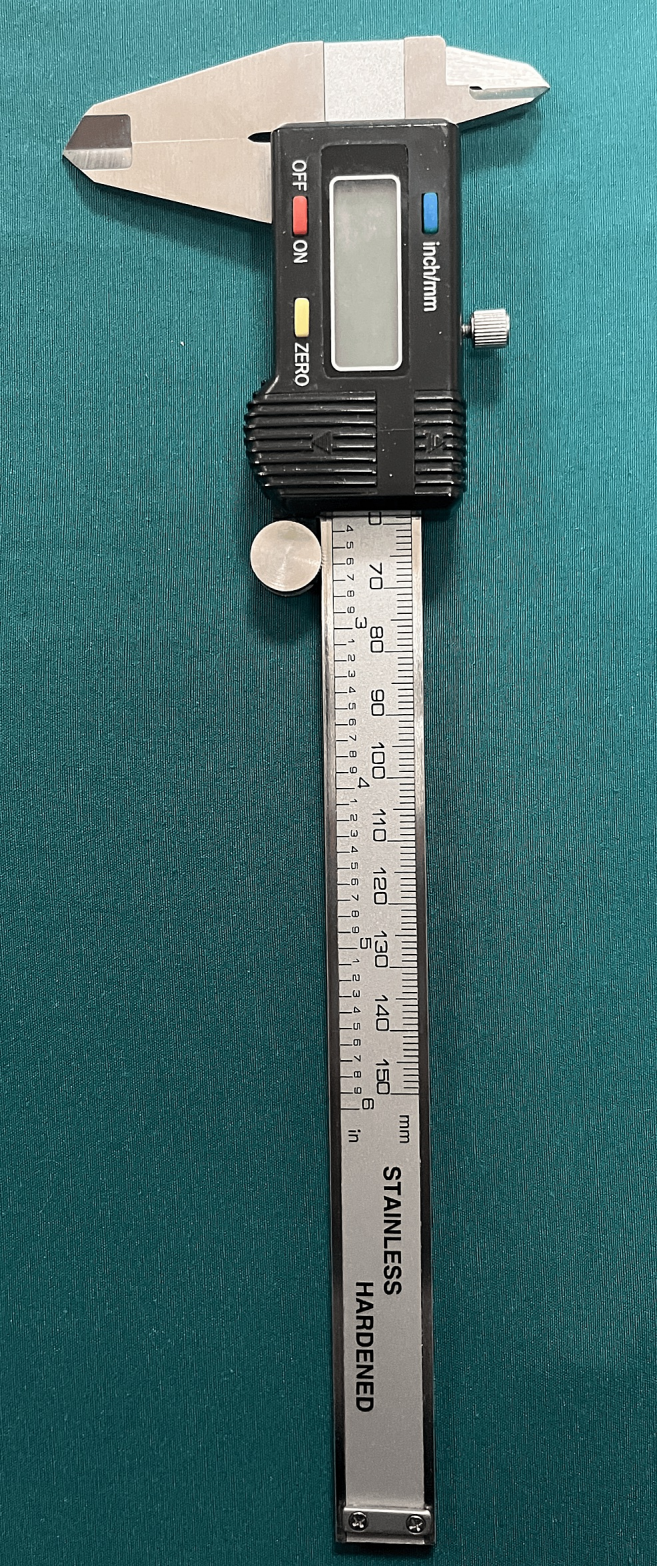
Demonstration of digital caliper

The digital caliper with an accuracy of 0.01 mm (150 mm length) was used for measurements (Figure 2). The measurements were done by the same person (MÜ) twice and averaged to avoid the intra observer error.

Statistical analysis

Statistical analysis was performed using SPSS version 23 (IMB Inc., Armonk, New York). The conformity of the data to the normal distribution was examined using histogram graphs and Kolmogorov-Smirnov/Shapiro-Wilk tests. A descriptive statistical analysis (percentage, mean, standard deviation, minimum and maximum values) of the parameters was done. Statistical differences between the right and left-sided tali were made using the Mann-Whitney U test. The Mann-Whitney U test for post hoc analysis was performed after the Kruskal Wallis test to compare the total number of foramina on each surface of tali. A p-value of <0.05 was accepted as statistically significant in all analyses.

## Results

The total number of VF on 62 tali was determined as 2161. While 1754 (81.17%) of all VF were observed on the neck, 407 (18.83%) were on the body. Type B calcaneal articular facet was observed in all dry tali. No VF was detected on the articular surfaces and head of the talus. There was no statistically significant difference between the right and left-sided talus for the number of VF (p>0.05; Tables 1, 2).

**Table 1:** Localization of the VF on the neck R - right; L - left; n - number; VF - vascular foramina

Surface	Side	Number of VF		Total VF, n (%)	Total, n (%)	p-value
		Min	Max			
Superior	R	3	15	234 (31.28)	578 (32.95)	0.903
	L	2	26	344 (34.19)		
Inferior	R	5	28	310 (41.44)	708 (40.36)	0.903
	L	5	16	398 (39.56)		
Medial	R	1	11	133 (17.78)	296 (16.88)	0.288
	L	1	11	163 (16.20)		
Lateral	R	0	6	71 (9.49)	172 (9.81)	0.948
	L	0	7	101 (10.04)		

**Table 2:** Localization of the VF on the body R - right; L - left; n - number; VF - vascular foramina

Surface	Side	Number of VF		Total VF, n (%)	Total, n (%)	p-value
		Min	Max			
Superior	R	0	0	0 (0)	0 (0)	1.000
	L	0	0	0 (0)		
Inferior	R	0	0	0 (0)	0 (0)	1.000
	L	0	0	0 (0)		
Medial	R	1	13	92 (53.80)	233 (57.25)	0.474
	L	1	10	141 (59.75)		
Lateral	R	0	4	21 (12.28)	47 (11.55)	0.839
	L	0	5	26 (11.02)		
Posterior	R	0	5	58 (33.92)	127 (31.20)	0.334
	L	0	4	69 (29.24)		

Neck

The VF on the neck of the talus was found to be most frequent (708; 40.36%) on the inferior surface and at least 172 (9.81%) on the lateral surface (Table 1). While there were VF on the superior, inferior, and medial surfaces of the neck of all talus, on seven (11.29%) tali, no VF was found on the lateral surface of the neck. A statistically significant difference was found between the number of VF and location on the surfaces of the neck (χ2(3) = 142.802, p=0.001, the mean rank was 159.71 on the superior surface, 192.46 on the inferior surface, 90.13 on the medial surface, 55.70 on the lateral surface). Comparing the frequency of the VF on the surfaces of the neck of the talus, the inferior surface was found to be a significantly more common location than the medial surface (U =255.50, p=0.001), superior surface (U =1275.00, p=0.001) and lateral surface (U =22.00, p=0.001).

Body

**Figure 3:**
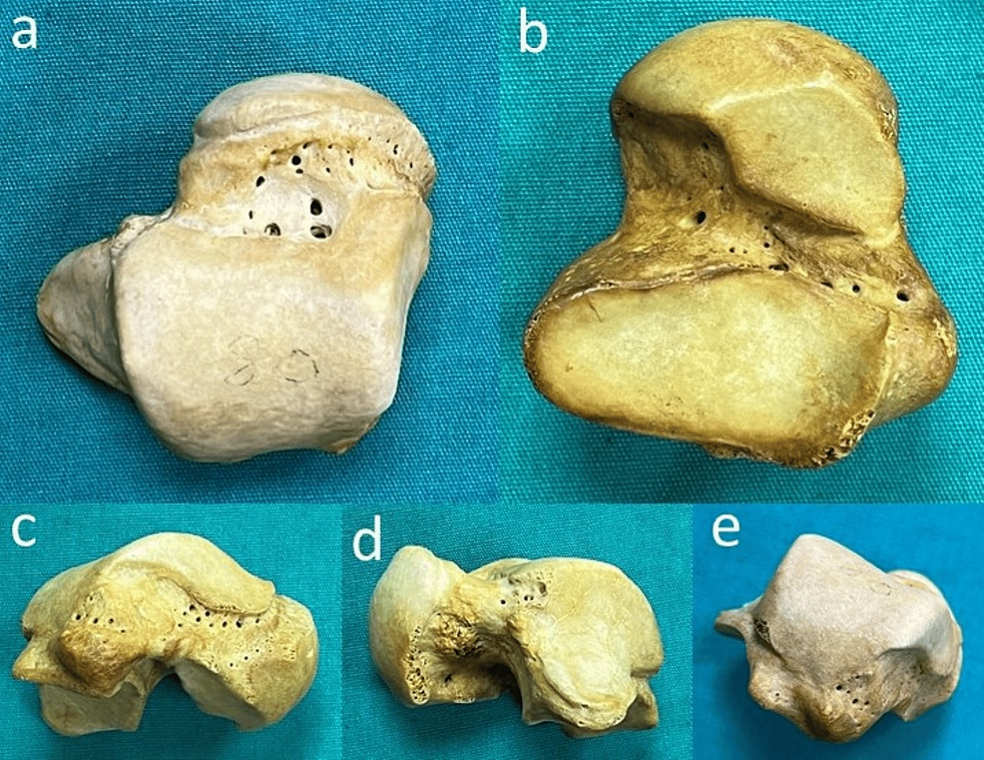
Demonstration of various localizations of VF of talus a) localization of VF on the superior surface of the neck; b) localization of VF on the inferior surface of the neck; c) localization of VF on the medial surface of the neck and body; d) localizations of VF on the lateral surface of neck and body; e) localization of VF on the posterior surface of the body. VF - vascular foramina

While the number of VF on the body of talus was found as 233 (57.25%) on the medial surface, 127 (31.20%) on the posterior surface, 47 (11.55%) on the lateral surface, no VF was observed on the superior and inferior surfaces of the body of talus (Table 2). Also, VF was not detected on the posterior surfaces and lateral surfaces of the body in 11.29% and 66.13% of all the tali, respectively. A statistically significant difference was found between the number of VF and location on the surfaces of the body of the talus (χ2(3) = 220.327, p=0.001, mean rank was found as 256.39 on the medial surface, 134.21 on the lateral surface, and 213.90 on the posterior surface). Comparing the frequency of the VF on the surfaces of the body of the talus, the medial surface was found to be a significantly more common location than the posterior surface (U=1048.00, p=0.001) and lateral surface (U=385.00, p=0.001). The various localizations of the VF on the talus are demonstrated in Figure 3.

While 1521 (70.38%) of these foramina were ≥0.6 mm, 640 (29.62%) were <0.6 mm. It was observed that 1290 (73.55%) of the foramina on the neck of the talus and 231 (56.76%) of the foramina on the body of the talus were ≥0.6 mm (Table 3).

**Table 3:** The size of the VF n - number; VF - vascular foramina

Surface		≥0.6 mm n (%)	<0.6 mm, n (%)
Neck	Superior	444 (76.82)	134 (23.18)
	Inferior	557 (78.67)	151 (21.33)
	Medial	186 (62.84)	110 (37.16)
	Lateral	103 (59.88)	69 (40.12)
	Total	1290 (73.55)	464 (26.45)
Body	Superior	0 (0)	0 (0)
	Inferior	0 (0)	0 (0)
	Medial	115 (49.36)	118 (50.64)
	Lateral	38 (80.85)	9 (19.15)
	Posterior	78 (61.42)	49 (38.58)
	Total	231 (56.76)	176 (43.24)
Total		1521 (70.38)	640 (29.62)

The mean values of the CFI, FFI, CFM, and FFM were measured at 21.42±2.89 mm, 42.98±4.95 mm, 16.48±4.13 mm and 31.36±4.86 mm, respectively. The mean value of TL was 55.14±4.69 mm, and ML was 49.18±4.18 mm. The mean values of the FI1, FI2, FI3 and FI4 were calculated 38.85±4.20%, 77.89±5.00%, 33.52±8.02% and 63.91±9.09%, respectively. There was no significant difference between the right and left side for measured parameters (Table 4).

**Table 4:** Morphometric properties of the VF VF - vascular foramina; TL - total length; ML - medial surface length; CFI - the closest foramina on the inferior surface; FFI - the furthest foramina on the inferior surface; CFM - the closest foramina on the medial surface; FFM - the furthest foramina on the medial surface; FI1 - the foraminal index 1; FI2 - the foraminal index 2; FI3 - the foraminal index 3; FI4 - the foraminal index 4

Parameter	Right	Left	Total	p value
TL (mm)	55.53±3.99	54.85±5.18	55.14±4.69	0.535
ML (mm)	50.17±3.79	48.47±4.36	49.18±4.18	0.104
CFI (mm)	21.66±3.24	21.25±2.64	21.42±2.89	0.503
FFI (mm)	43.36±4.31	42.71±5.41	42.98±4.95	0.471
CFM (mm)	16.60±4.35	16.39±4.03	16.48±4.13	0.700
FFM (mm)	30.66±4.36	31.86±5.19	31.36±4.86	0.494
FI1 (%)	38.97±4.81	38.77±3.77	38.85±4.20	0.549
FI2 (%)	78.03±4.26	77.79±5.53	77.89±5.00	0.943
FI3 (%)	33.17±8.64	33.77±7.65	33.52±8.02	0.954
FI4 (%)	61.35±8.99	65.75±8.82	63.91±9.09	0.101

## Discussion

The distal and plantar surfaces of the head of the talus have articular surfaces for navicular bone and calcaneus, respectively. The neck of the talus has sulcus tali on the plantar surface; when the talus and calcaneus articulate, the sulcus tali and the sulcus calcanei together form the tarsal sinus [1]. On the body of the talus, the lateral articular surface articulates with the lateral malleolus, the medial articular surface articulates with the medial malleolus, and the trochlear surface articulates with the distal part of the tibia, superiorly and named as the talar dome. The body has two processes (posterior and lateral processes). On the posterior process, there is also a longer lateral tubercle and a less prominent medial tubercle [11]. Up to 70% of the surface of the talus is covered by articular cartilage [12]. The articular surfaces lack lymphatic and blood vessels since the talus has few non-articular regions, the blood supply and healing of the talus are limited [9,13].

The branches of the dorsalis pedis artery supply the superior neck of the talus, and the anastomosis of the tarsal canal and tarsal sinus arteries provides an arterial supply of the inferior neck [8]. The blood supply of the head of the talus is provided by branches of the dorsalis pedis artery medially and branches from the anastomosis of the tarsal canal and tarsal sinus arteries laterally [11]. The body of the talus is also supplied by the tarsal sinus and dorsalis pedis arteries [14].

The talus is the second most common fractured tarsal bone, following the calcaneus, and constitutes 0.1% to 2.5% of all fractures and 3% to 5% of ankle and foot fractures. Talus neck fractures constitute approximately 50% of talus fractures and are the most common site of talus fractures [15,16]. Also, fractures of the head of the talus are the least common region of talus fractures, which represent approximately 3% to 10% of talus fractures [15]. Although talus fractures are rare, talus is more prone to osteonecrosis than other bones due to its poor arterial supply [9]. Osteonecrosis may occur due to traumatic and non-traumatic causes and is more common due to traumatic causes [17]. The incidence of osteonecrosis in talus neck fractures is higher due to the high number of blood vessels feeding the bone in the neck region and the deterioration of blood flow as a result of the fracture [15]. In accordance with this situation, the majority of the VF (1754; 81.17%) were detected on the neck region in this study.

In this study, VF was detected only on non-articular surfaces of the talus, similar to previous studies [8,18]. We observed no VF on the head of the talus. While the VF was mostly found on the neck of the talus, it was mostly located on the inferior surface of the neck in this study, similar to Vani et al. [8]. Also, Oppermann et al. studied VF of the talus with plastination samples, and they found VF was mostly located on the inferior surface of the neck, which was in accordance with our study [19].

Knowing the variations of the calcaneal and talar articular surfaces of the talus and calcaneus is important in the treatment of diseases such as pes planus, valgus deformities, talocalcaneal arthritis, and subtalar instability [20]. In previous studies, type B talus was detected mostly, and in our study, all talus were Type B [10,21,22]. In a study by Vani et al., no significant difference was found between the joint types and the number of VF in the Indian population [8]. Statistical analysis between joint types and VF could not be performed in this study since all tali were detected as type B.

Vani et al. used Kirschner wire (0.5 mm) to evaluate the diameter of the VF, and the majority of VF (1427; 77.05%) were ≥0.5 mm [8]. We evaluated the diameter of the VF with gauge size 26 hypodermic needle (0.6 mm), and similar to Vani et al., the majority of VF (1521; 70.38%) were ≥0.6 mm in this study.

In this study, according to Vani et al., the lateral tubercle of the posterior process was used as a reference point for the foraminal index of the VF on the inferior sure, face but unlike the study of Vani et al., the medial tubercle of the posterior process was used as a reference point for the foraminal index of the VF on the medial surface of the talus. It is thought that the medial tubercle is a more appropriate anatomical point to calculate the foraminal index of the VF on the medial surface than the lateral tubercle. Vani et al. found that FI1 was 47.90% and FI2 was 75.08% [8]. In our study, FI1 was 38.85%, and FI2 was 77.89%; the FI1 value was lower than Vani et al., while the FI2 value was found to be compatible [8]. The foraminal index of the VF on the medial and inferior surfaces of the talus is important to know the localization of the VF. This morphological data will be helpful in reducing the damage to the blood supply of the talus during surgical approaches to the talus.

Talus fractures are operated with anterolateral, anteromedial, lateral, anterior, extended anterolateral, posterolateral, and posteromedial routes and medial, fibular, and bilateral osteotomies [12]. Since VF was least common on the lateral surfaces of the neck and body in this study, lateral approaches to the talus may be safer than other approaches for minimizing vascular damage.

Limitations of the study

This study has some limitations. The age and sex of the bones were unknown; therefore, age and sex differences of VF were not evaluated. Also, the sample size was limited to 62 tali. Further studies should be performed with a larger sample size and bones with known age and sex.

## Conclusions

We observed that VF are usually localized to the inferior surface of the neck, and most of them were ≥0.6 mm. No VF was detected on the head of the talus, as well as on the superior and inferior surfaces of the body of the talus. Since VF were observed least common on the lateral surfaces of the neck and body, lateral approaches to the talus may be safer than other approaches for minimizing vascular damage. To our knowledge, there is no study about the morphology of VF of the talus in Türkiye samples. We believe that the results of this study will provide reference data for morphology and morphometry of VF of talus.
